# Sociodemographic determinants and mortality of premature newborns in a medium and low-income population in Colombia, 2017-2019

**DOI:** 10.7705/biomedica.6863

**Published:** 2023-09-30

**Authors:** Javier Torres-Muñoz, Daniel Alberto Cedeño, Jennifer Murillo, Sofía Torres-Figueroa, Julián Torres-Figueroa

**Affiliations:** 1 INSIDE Research Group, Departmento de Pediatría, Universidad del Valle, Cali, Colombia, Universidad del Valle INSIDE Research Group Departmento de Pediatría Universidad del Valle Cali Colombia; 2 Universidad del Valle, Departmento de Pediatría, Cali, Colombia Universidad del Valle Universidad del Valle Departmento de Pediatría Cali Colombia; 3 Facultad de Ciencias de la Salud, Universidad ICESI, Cali, Colombia Universidad Icesi Facultad de Ciencias de la Salud Universidad ICESI Cali Colombia

**Keywords:** Obstetric labor, premature, social determinants of health, developing countries, Colombia, trabajo de parto prematuro, determinantes sociales de la salud, países en desarrollo, Colombia

## Abstract

**Introduction.:**

The birth of premature babies is a public health problem with a high impact on infant morbidity and mortality. About 40% of mortality in children under five years occurs in the first month of life.

**Objective.:**

To identify the association between maternal sociodemographic factors, premature birth, and mortality in newborns under 37 weeks in Santiago de Cali, 2017-2019.

**Materials and methods.:**

We conducted a descriptive, cross-sectional study. We evaluated the records of Cali’s Municipal Public Health Office. We calculated the crude and adjusted odd ratios and confidence intervals (95%) using the logistic regression model, data processing in Stata 16, and georeferencing the cases in the QGIS software.

**Results.:**

From 2017 to 2019, premature babies in Cali corresponded to 11% of births. Poor prenatal care increased 3.13 times the risk of being born before 32 weeks (adjusted OR = 3.13; 95% CI = 2.75 - 3.56) and 1.27 times among mothers from outside the city (adjusted OR = 1.27; 95% CI = 1.15-1.41). Mortality was 4.29 per 1,000 live births. The mortality risk in newborns weighing less than 1,000 g increased 3.42 times (OR = 3.42; 95% CI = 2.85-4.12), delivery by cesarean section in 1.46 (OR = 1.46; CI 95% = 1.14-1.87) and an Apgar score - five minutes after birth- lower than seven in 1.55 times (OR = 1.55; CI 95% = 1.23-1.96).

**Conclusions.:**

We found that less than three prenatal controls, mothers living outside Cali, afro-ethnicity, and cesarean birth were associated with prematurity of less than 32 weeks. We obtained higher mortality in newborns weighing less than 1,000 g.

## Resumen

**Introducción.:**

El nacimiento de bebés prematuros es un problema de salud pública con gran impacto en la morbimortalidad infantil: cerca del 40 % de las muertes de niños menores de cinco años sucede en el primer mes de vida.

**Objetivo.:**

Identificar la asociación entre los factores sociodemográficos maternos, el parto prematuro y la mortalidad en recién nacidos menores de 37 semanas en Santiago de Cali, 2017-2019.

**Materiales y métodos.:**

Se hizo un estudio descriptivo transversal. Se evaluaron los registros de la Secretaría de Salud Pública Municipal de Cali. Se calcularon las razones de probabilidad y los intervalos de confianza (95 %) crudos y ajustados mediante el modelo de regresión logística, en tanto que los datos se procesaron en Stata 16 y los casos se georreferenciaron con el programa QGIS.

**Resultados.:**

Entre el 2017 y el 2019, los nacimientos de bebés prematuros en Cali correspondieron al 11 %. El control prenatal deficiente aumentó 3,13 veces el riesgo de nacer con menos de 32 semanas (OR ajustado = 3,13; IC_95%_ = 2,75-3,56) y, en madres de municipios fuera de la ciudad, 1,27 veces (OR ajustado = 1,27; IC_95%_ = 1,15-1,41). La mortalidad fue de 4,29 por 1.000 nacidos vivos. Nacer con un peso menor de 1.000 g aumentó el riesgo de mortalidad en 3,42 veces (OR = 3,42; IC_95%_ = 2,85-4,12) y, un puntaje Apgar menor de siete a los cinco minutos del nacimiento, en 1,55 veces (OR=1,55; IC_95%_ = 1,23-1,96).

**Conclusiones.:**

Se encontró que tener menos de tres controles prenatales, la procedencia de la madre fuera de Cali, ser afrodescendiente y el parto por cesárea, estaban asociados significativamente con la prematuridad de menos de 32 semanas. Hubo mayor mortalidad en los recién nacidos con menos de 1.000 gramos al nacer.

The birth of premature babies is a public health problem with a high impact on infant morbidity and mortality ^1^. In 2018, about 40% of mortality in children under five years occurred in their first month of life, mainly associated with prematurity factors ^(^^2^, which explains why the neonatal period is critical and determining in children’s health.

According to the World Health Organization (WHO), preterm infants are classified according to their gestational age ^3^ into “extreme preterm” (less than 28 weeks), “early preterm” (between 28 and 31.6 weeks), “moderate preterm” (between 32 and 33.6 weeks), and “late preterm” (between 34 and 36.6 weeks). They can also be classified according to their birth weight as “low birth weight” (less than 2,500 g), “very low birth weight” (less than 1,500 g), and “extremely low birth weight” (less than 1,000 g). Higher prematurity and lower birth weight increase the risk of neonatal mortality and morbidity, as well as the presence of pathologies such as respiratory distress syndrome, bronchopulmonary dysplasia, intraventricular hemorrhage, necrotizing enterocolitis, neonatal sepsis, perinatal asphyxia and cerebral palsy, among others ^2^.

Premature infants present difficulties during their neonatal stage and in the long term, affecting child, adolescent, and adult health by increasing the risk of neurological, visual, and auditory alterations ^4^. In adults, prematurity can lead to a greater risk of developing metabolic syndrome ^5^ and chronic kidney disease ^6^, among other comorbidities.

In 2014, based on national civil registration and vital statistics databases, the WHO established an estimated rate of premature births worldwide of 10.6% (14,835,606 premature births per 139,945,950 live births). The highest rate of premature births was found in North Africa (13.4%), while the rate in Latin America and the Caribbean was reported at 9.8%. The countries with the highest proportion of premature births were India (18.5%), China (12%), and Nigeria (5%) ^1^. In Latin America and Caribbean countries, the proportion of extremely preterm infants was 8.9% (4.1% worldwide) of early preterm infants, 9.8% (11.3% worldwide), and of moderate and late premature infants, 81.2% (84.7% globally). The highest proportion of extremely preterm infants worldwide was reported in Latin America and the Caribbean ^1^.

According to the Colombian *Instituto Nacional de Salud*, from 2007 to 2016, the prematurity rate in Colombia was 9.07%, in conformity with the last decade’s trend ^7^. In 2017, the WHO reported neonatal mortality worldwide of almost 2.5 million children ^8^^,^^9^. About one million babies die each year due to prematurity complications ^10^^,^^11^, considered the second most common cause of death in children under five years ^3^^,^^12^, especially in low- and middle-income countries where it is associated with lack of availability and access to services and care personnel for premature infants ^3^.

In 2018, this indicator showed 7.7 cases per 1,000 live births in Latin America. In 2019, neonatal mortality in Colombia was 15.2 deaths per 1,000 live births ^13^^,^^14^, while the mortality rate among preterm newborns in Valle del Cauca was 12 per 1,000 live births and 13.9 per 1,000 live births in Cali ^(15)^.

Risk factors associated with premature births include sociodemographic factors (maternal age, race, socioeconomic level, marital status, other); maternal biological conditions (uterine malformations, maternal pathological history, other); behavioral factors (smoking, alcohol and psychoactive substances consumption, malnutrition, absence or poor prenatal controls), and complications during pregnancy (hypertensive disorders, multiple pregnancies, amniotic fluid disorders, fetal anomalies, hemorrhages, infections, other) ^2^^,^^16^.

Here we describe the sociodemographic factors associated with premature births and mortality in low- and middle-income populations in Cali, Colombia.

## Materials and methods

### Definitions

According to WHO, premature birth occurs before 37 weeks of gestation or less than 259 days from the first day of a woman’s last menstrual period ^1^, and perinatal mortality is death after 22 weeks of gestation or a fetal weight of 500 g or more up to seven days after birth. Neonatal mortality is death between the birth day and the first 28 days of life ^9^. The incidence of preterm birth was calculated as the ratio of the preterm live births divided into the total live births, and the incidence of premature mortality, as the ratio of preterm infants who died divided into the preterm infants alive. Inadequate prenatal control was defined as assistance to three or fewer controls ^16^.

### Type of study

We conducted a descriptive, observational, cross-sectional study based on data from the public health surveillance system of births of at the *Secretaría de Salud Pública Municipal* in Cali (Colombia) from 2017 to 2019.

### Study area

The study was based on the reports from Cali´s *Secretaría de Salud Pública Municipal*, which are mandatory for the health institutions in the city.

### Study population

We included babies born prematurely under 37 weeks of gestational age who complied inclusion criteria: Cali’s premature newborns (less than 37 weeks of gestation) registered with the *Departamento Administrativo Nacional de Estadística* (DANE) between January 2017 and December 2019. The exclusion criteria included term newborns (37 or more weeks of gestation); newborns not registered with the *Secretaría de Salud Pública Municipal* and born before January 2017 or after December 2019; preterm newborns with a weight for a gestational age above the 97th percentile (Fenton tables).

### Sample size

**Figure 1:**
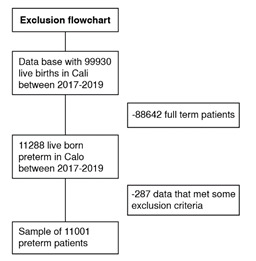
Diagram of the selected preterm infants included in the study between 2017 and 2019

We considered all premature births recorded by the *Secretaría de Salud Pública Municipal* between January 2017 and December 2019, according to the inclusion and exclusion criteria. There were 3,716 registrations in 2017, 3,646 in 2018, and 3,639 in 2019, for a total of 11,001 records (figure 1).

### Data analysis

First, we made a univariate analysis exploring the distribution of continuous variables and frequency distributions in qualitative variables, data loss, and consistency of information. We measured frequency, central tendency, and dispersion according to each variable’s classification and distribution.

Subsequently, we made a bivariate analysis to compare the distribution of the characteristics of interest using chi square and Fischer’s exact tests. For this bivariate analysis, prematurity was categorized according to the WHO classification of gestational age (up to 32 weeks and between 33 and 37 weeks).

Finally, the odd ratio strength of association and the respective confidence intervals (95% CI) were determined by logistic regression. Data analysis and processing were done with Stata, version 16, and case georeferencing with the QGIS Software.

### Ethical statement

The study was conducted according to the guidelines of the Declaration of Helsinki. It was approved by the institutional ethics committee at Cali’s *Secteraría de Salud Pública Municipal* (code E004-021) and by the Ethics Committee at the *Universidad del Valle* (code E004-021).

## Results

From 2017 to 2019, the DANE reported 99,930 births in Cali, and 11% (11,001) were premature births (less than 37 weeks at birth) (figure 1). Out of these, 6,672 (60.7%) mothers lived in Cali, while 4,017 (36.5%) were from other cities, and 312 (2.9%) had no information recorded for the place of residence.

Regarding maternal variables, 1,929 (17.5%) subjects were Afro-descendants; 910 (8.3%) were adolescent mothers; 1,624 (14.8%) were over 35 years; 1,774 (16.3%) were mothers without a partner; 871 (8.0%) had completed primary school; 4,717 (42.9%) were new mothers, and 1,734 (15.8%) had poor prenatal controls. Regarding newborns variables, 6,007 (54.6%) were delivered by cesarean section; 1,413 (12.8%) were products of twin pregnancies, and 5,861 (53.3%) were male.

According to the WHO classification of prematurity, extreme prematurity was found in 613 (5.6%) newborns, early prematurity in 1,227 (11.2%), moderate prematurity in 1,299 (11.8%), and late prematurity in 7,862 (71.5%). Regarding birth weight, 714 (6.5%) newborns were less than 1,000 g; 1,025 (9.3%) were between 1,000 and 1,500 g; 5,385 (49%) were between 1,500 and 2,500 g, and 3,877 (35.2%) weighed more than 2,500 g. Of the premature newborns, 8,016 (72.9%) were delivered at private institutions.

Prematurity under 32 weeks occurred in 1,840 (16.7%) newborns. In this group, 829 (45.0%; p<0.00) newborns’ mothers came from municipalities outside Cali; 492 (26.7%; p<0.05) had poor prenatal controls; 1,482 (80.5%; p<0.05) born at private institutions; 1,319 (71.7%; p<0.05) were delivered by cesarean section; 375 (20.4%; p<0.05) were Afro-descendant; 281 (15.3%; p<0.05) were products of twin pregnancies, and 406 (22.0%; p<0.05) died (table 1).

**Table 1:** Premature infants’ characteristics when comparing them by gestational age: less than 32 weeks versus more than 32 weeks

Characteristics		Gestational age			p value
		< 32 weeks n = 1,840 (%)		≥ 32 weeks n = 9,161 (%)	
Birth area	Other municipalities	829	(45.00)	3,500 (38.20)	< 0.001
	Cali	1,011	(55.00)	5,661 (61.80)	
Birth site	Health institution	1,829	(99.40)	9,127 (99.63)	0.252*
	Home	6	(0.33)	23 (0.25)	
	Other	5	(0.27)	11 (0.12)	
Institution type	Public	358	(19.46)	2,627 (28.68)	< 0.001
	Private	1,482	(80.54)	6,534 (71.32)	
Newborn sex	Male	998	(54.24)	4,863 (53.10)	0.357
	Female	841	(45.71)	4,296 (46.90)	
Newborns’ weight (g)	< 1,000	702	(38.15)	12 (0.13)	< 0.001*
	1,000-1,500	760	(41.30)	265 (2.89)	
	>1,500-2,500	378	(20.54)	5,007 (54.66)	
	< 2,500	0	(0.00)	3,877 (42.32)	
Number of prenatal controls	≤ 3	492	(26.74)	1,242 (13.56)	< 0.001
	≥ 4	1,348	(73.26)	7,918 (86.44)	
Delivery method	Vaginal delivery	521	(28.32)	4,473 (48.83)	< 0.001
	Cesarean section	1,319	(71.68)	4,688 (51.17)	
Twin pregnancy	Yes	281	(15.27)	1,132 (12.36)	<0.001
	No	1,559	(84.73)	8,029 (87.64)	
Apgar score at five minutes	< 7	761	(41.54)	5,626 (61.61)	< 0.001
	8 - 10	1,071	(58.46)	3,505 (38.39)	
Ethnicity	Afrodescendant	375	(20.38)	1,554 (16.96)	< 0.001
	Others	1,465	(79.62)	7,607 (83.04)	
Maternal age (years)	< 19	144	(7.83)	766 (8.36)	0.741
	19 - 35	1,425	(77.45)	7,042 (76.87)	
	> 35	271	(14.73)	1,353 (14.77)	
Marital status	With a partner	1,524	(83.83)	7,567 (83.64)	0.844
	Without a partner	294	(16.17)	1,480 (16.36)	
Educational level	Primary	137	(7.67)	734 (8.12)	0.525
	Secondary and superior	1,649	(92.33)	8,307 (91.88)	
Number of pregnancies	1	779	(42.34)	3,938 (42.99)	0.475
	2 - 4	854	(46.41)	4,279 (46.71)	
	> 4	207	(11.25)	944 (10.30)	
Social security	Subsidized	785	(42.66)	3,989 (43.54)	0.785
	Contributory	968	(52.61)	4,747 (51.82)	
	Uninsured	87	(4.73)	425 (4.64)	
Nationality	Colombian	1,840	(100.00)	9,159 (99.98)	0.526*
	Migrants	0	(0.00)	2 (0.02)	
Mortality	Yes	406	(22.07)	3 (0.03)	< 0.001*
	No	1,434	(77.93)	9,158 (99.97)	

Poor prenatal controls increased 3.13 times the risk of being born at less than 32 weeks (ORa = 3.13; CI 95% = 2.75-3.56); being an Afro-descendant newborn, 1.31 times (ORa = 1.31; CI 95% = 1.15-1.50), and the mother coming from municipalities other than Cali, 1.27 times (ORa = 1.27; CI 95% = 1.15-1.41). Conversely, births in private institutions (ORa = 0.56; 95% CI = 0.49-0.64) and births by cesarean section (ORa = 0.39; 95% CI = 0.35-0.44) were protective factors (table 2).

**Table 2:** Multivariate analysis of the significant variables for less than 32 weeks and more than 32 weeks of gestational age at birth

	Gestational age ≥ 32 week		Gestational age < 32 week	
Variable	OR (95% CI)	p value	OR (95% CI)	p value
Type of institution	0.60 (0.53-0.68)	< 0.001	0.56 (0.49-0.64)	< 0.001
Number of prenatal controls	2.32 (2.06-2.62)	< 0.001	3.13 (2.75-3.56)	< 0.001
Delivery method	0.41 (0.37-0.46)	< 0.001	0.39 (0.35-0.44)	< 0.001
Twin pregnancy	1.27 (1.10-1.47)	0.001	1.04 (0.90-1.21)	0.550
Ethnicity	1.25 (1.10-1.42)	< 0.001	1.31 (1.15-1.50)	< 0.001
Other municipalities	1.32 (1.19-1.46)	< 0.001	1.27 (1.15-1.41)	< 0.001

OR: odds ratio; CI: confidence interval

Mortality was 4.29 per 1,000 live births. Eighty-one (18.9%) corresponded to Afro-descendant newborns; 29 (6.8%) were born from adolescents mothers; 27 (6.3%) from mothers without a partner; 24 (5.8%) from mothers with only primary education; 176 (41.0%) from new mothers; 158 (36.8%) had poor prenatal controls; 280 (65.3%) were delivered by cesarean section; 81 (18.9%) were products of twin pregnancies, and 255 (59.8%) were male. When categorizing mortality by gestational age, 305 (71.1%) corresponded to premature babies under 28 weeks, 101 (23.5%) from 28 to 32 weeks, 18 (4.2%) from 32 to 34 weeks, and 5 (1.2%) from 34 to 37 weeks. Four hundred and six (406; 94.6%) children under 32 weeks died. Regarding the weight, 373 were born below 1,500 g, representing 86.9% of the deceased (table 3).

**Table 3:** Premature mortality in Cali, Colombia, according to premature newborns’ year of birth, gestational age, and birth weight

Variable		Mortality (n)	Total (n)	%
Year	2017	158	3,716	4.3
	2018	143	3,646	3.9
	2019	128	3,639	3.5
Accumulated		429	11,001	3.9
Gestational age (weeks)				
	< 28	305	613	71.09
	28-32	101	1,227	23.54
	32-34	18	1,299	4.19
	34-37	5	7,862	1.17
Weight (g)				
	<1,000	285	714	66.43
	1,000-1,500	88	1,025	20.51
	1,500-2,500	54	5,385	12.58
	>2,500	2	3,877	0.48

We found a significant association between higher mortality and gestational age less than 32 weeks in 94.6% (p<0.01) of the children. The weight was less than 1,000 g in 66.4% (p<0.01), 59.4% (p<0.01) were males, 65.2% (p<0.01) were born by cesarean section, and the Apgar score was less than seven at five minutes after birth in 53.8% (p<0.05). In the group with more than three prenatal controls, 63.2% (p<0.01) had a higher mortality rate.

In the final model, the mortality risk of newborns weighing less than 1,000 g increased 3.42 times (OR = 3.42; 95% CI = 2.85-4.12). In babies born under 32 weeks, that risk increased 19.92 times (OR = 19.68; CI 95% = 11.77-33.72); being male, 1.50 times (OR = 1.50; CI 95% = 1.19-1.90); born by cesarean section, 1.46 times (OR = 1.46; CI 95% = 1.14-1.87), and an Apgar score below seven at five minutes after birth, 1.55 times (OR = 1.55; CI 95% = 1.23-1.96) (table 4).

**Table 4:** Significant variables associated with premature newborns’ mortality born under 37 weeks

Mortality	p value	OR	95% IC
Birth weight < 1,000 g	0.00	3.42	2.85-4.12
Gender (male)	0.00	1.50	1.19-1.90
Delivery method (cesarean)	0.00	1.46	1.14-1.87
Gestational age of the newborn (< 32 weeks)	0.00	19.92	11.77-33.72
Apgar score at five minutes of birth (< 7)	0.00	1.55	1.23-0.96

OR: odds ratio; CI: confidence interval

**Figure 2:**
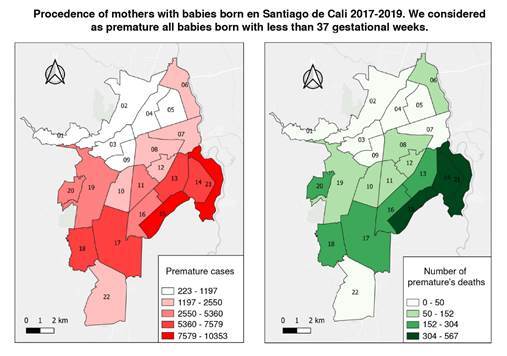
Left: Premature cases by mothers’ commune origin. Right: Death rates in premature infants by mothers’ commune origin. Distribution of the 6,672 mothers of babies born under 37 weeks, residing in Cali, with a mortality of 72.49% (n=311) of the total mortality (n=429). Of the 6,672 mothers, 33.38% (n=2,227) came from the city east communes (13, 14, 15, and 21), with a mortality rate of 33.76% (n=105). Mothers from other municipalities were 39.35% (n=4,329) and the mortality rate among their babies was 27.50% (n=118).

The georeferencing of premature infants of mothers from Cali (6,672) identified a non-significant number of premature infants (33.4%; n=2,227) and mortality rates (33.76%; n=105) in communes 13, 14, 15, and 21, in the east of Cali (figure 2). Inadequate prenatal control (16.9%) in mothers from other municipalities and the risk of delivering babies before 37 weeks were significantly higher compared to Cali´s risk (14.9%; p<0.01). The mortality of babies born to mothers from these municipalities was 27.50% (n=118).

## Discussion

According to our study, the prematurity rate in Cali was slightly higher than that reported for Colombia, other Latin American countries, and the world ^15^. A significant number of premature births occurred among mothers from low-income families living in eastern communes of the city (33.4%; 2,227), representing 24.3% of Cali’s houses and 15.2% of its total area. Low income was defined according to the city’s multidimensional poverty analysis, issued by multiple government entities ^17^^,^^18^. This analysis identified 36.3% of people living in monetary poverty in 2020 in Cali (36 out of 100 people were poor, more than in 2019 by 14.4 percentage points). Such an indicator was higher in women, with 1.9 percentage points above men ^17^. Belonging to an Afro-ethnic group increased the gap from 10% to 30% ^18^.

All these factors reveal this population vulnerability, characterized by high social inequalities and unsatisfied basic needs. Their association with premature birth and its complications has already been raised by other authors, who found significant relations and differences between birth rates, survival of premature babies, place of origin, poverty, nutritional deficiencies (lack of iron, calcium, and vitamins), and difficulties to access health services ^19^^,^^20^.

When we evaluated the maternal variables by gestational age, we found that babies under 32 weeks have a three-fold risk of not having adequate prenatal care compared to those over 32 weeks. In 2013, a study conducted among late preterm infants in a public institution in Cali found that in 98% of the cases, the mothers had inadequate prenatal care (defined as fewer than three controls) ^21^. Another study in six low- and middle-income countries found that less than three prenatal controls increased the risk of premature birth by 1.68 times ^16^. A cohort study in Israel also showed that the lack of prenatal control and adverse pregnancy outcomes were significantly associated with recurrent preterm birth (up to four times in the first case) ^19^. Finally, a systematic review published in 2021 found that interventions for a better quality of prenatal care in low- and middle-income countries led to significant reductions in premature birth and fetal deaths ^2^.

Regarding ethnicity, we found that 17.5% of all premature births corresponded to the Afro-descendant population. The probability of being born within less than 32 weeks and belonging to the Afro-descendant ethnic group was 31% higher than in other ethnic groups. Publications evaluating ethnicity in the Unites States and the United Kingdom have found that African American and Afro-Caribbean women are at increased risk of preterm birth, with 16-18% in black women compared to 5-9% in white women. Black women are three to four times more likely to have preterm labor than women of other races or ethnic groups ^22^^,^^23^. A meta-analysis and systematic review of forty-five studies evaluating the effect of maternal ethnicity (African/ Black, Asian, Hispanic, other) on the risk of preterm birth showed that black ethnicity was associated with an increased risk of having a two-time higher rate of preterm birth compared to white mothers ^24^.

In our study, twin pregnancy appeared to be significant, but this was not evident when we adjusted the analysis.

Premature birth occurs due to early labor induction or delivery by cesarean section for medical or non-medical reasons. Some studies reported that unnecessary cesarean sections increased two-fold the probability of iatrogenic prematurity ^25^^,^^26^. An increased rate of cesarean sections is related to maternal age over 35 years and overweight ^27^. In our study, those under 32 weeks had a 61% more chance of being born by cesarean section.

The proportion of premature births within less than 32 weeks reported in this study was 16.7%, like the reported worldwide (16.0%) ^28^^,^^29^.

A 4.29 per 1,000 live births mortality rate was reported among premature newborns. Twenty-four-point five percent were born to mothers living east of the city. Newborns under 32 weeks and those weighing less than 1,000 g had a significantly higher probability of dying. Similar results were published in a multicenter population study conducted in Hubei Province, China, from 2001 to 2012 ^30^, where shorter gestation, lower birth weight, and lower income were associated with a higher mortality rate. The mortality rate was 13.4 per 1,000 live births, higher than in our study.

A study in 26 centers in six South American countries ^31^ found a birth mortality rate of 22.3% in premature infants of less than 37 weeks of gestational age, higher than in our study (4.29 per 1,000 live births). However, there was a high variability between centers. The study concluded that social determinants, lack of access to healthcare, gestational age less than 32 weeks, and low Apgar impact the mortality rates, similar to our results. There are significant variations in preterm birth rates and mortality between and within countries. Yet, the burden of preterm birth is particularly high in low- and middle-income countries, especially in Southeast Asia and Sub-Saharan Africa ^32^^-^^34^.

Our results will contribute to reduce the information gap about premature births in countries with medium and low-income populations like ours since 90% of publications report data from medium or high-income regions representing the lowest proportion of births in the world ^1^.

The strengths of our study included a database collecting information on the total number of births in Cali with a significant sample size of premature infants from different public and private institutions in the city. The limitations included incomplete information, specifically, the place of origin of the mothers (2.9% of the cases), introducing information bias. There was no information on the method used to evaluate gestational age, considering that WHO recommends prenatal ultrasound (before 24 weeks) for all pregnant women.

We found that less than three prenatal controls, mothers living outside Cali, afro ethnicity, and cesarean birth were significantly associated with newborns within less than 32 weeks and weighing under 1,000 g, implying higher mortality rates. We concluded that social determinants and lack of access to care impact premature births and infant mortality in low- and middle-income countries like Colombia, for which further studies are required.
